# Decision Support System for the Response to Infectious Disease Emergencies Based on WebGIS and Mobile Services in China

**DOI:** 10.1371/journal.pone.0054842

**Published:** 2013-01-23

**Authors:** Ya-pin Li, Li-qun Fang, Su-qing Gao, Zhen Wang, Hong-wei Gao, Peng Liu, Ze-rui Wang, Yan-li Li, Xu-guang Zhu, Xin-lou Li, Bo Xu, Yin-jun Li, Hong Yang, Sake J. de Vlas, Tao-xing Shi, Wu-chun Cao

**Affiliations:** 1 State Key Laboratory of Pathogens and Biosecurity, Beijing Institute of Microbiology and Epidemiology, Beijing, People’s Republic of China; 2 Institute of Command Automation, PLA University of Science and Technology, Nanjing, People’s Republic of China; 3 Department of Public Health, Erasmus Medical Center (MC), University Medical Center Rotterdam, Rotterdam, The Netherlands; INSERM & Universite Pierre et Marie Curie, France

## Abstract

**Background:**

For years, emerging infectious diseases have appeared worldwide and threatened the health of people. The emergence and spread of an infectious-disease outbreak are usually unforeseen, and have the features of suddenness and uncertainty. Timely understanding of basic information in the field, and the collection and analysis of epidemiological information, is helpful in making rapid decisions and responding to an infectious-disease emergency. Therefore, it is necessary to have an unobstructed channel and convenient tool for the collection and analysis of epidemiologic information in the field.

**Methodology/Principal Findings:**

Baseline information for each county in mainland China was collected and a database was established by geo-coding information on a digital map of county boundaries throughout the country. Google Maps was used to display geographic information and to conduct calculations related to maps, and the 3G wireless network was used to transmit information collected in the field to the server. This study established a decision support system for the response to infectious-disease emergencies based on WebGIS and mobile services (DSSRIDE). The DSSRIDE provides functions including data collection, communication and analyses in real time, epidemiological detection, the provision of customized epidemiological questionnaires and guides for handling infectious disease emergencies, and the querying of professional knowledge in the field. These functions of the DSSRIDE could be helpful for epidemiological investigations in the field and the handling of infectious-disease emergencies.

**Conclusions/Significance:**

The DSSRIDE provides a geographic information platform based on the Google Maps application programming interface to display information of infectious disease emergencies, and transfers information between workers in the field and decision makers through wireless transmission based on personal computers, mobile phones and personal digital assistants. After a 2-year practice and application in infectious disease emergencies, the DSSRIDE is becoming a useful platform and is a useful tool for investigations in the field carried out by response sections and individuals. The system is suitable for use in developing countries and low-income districts.

## Introduction

For years, emerging infectious diseases (EIDs) have appeared worldwide and not only threatened the health of people, but also a significant burden on global economic and public health [1], [2]. The emergence and spread of an outbreak of an infectious disease is usually unforeseen and result from complex, dynamic in which biological, social, ecological, and technological processes interconnect [3]. With the outbreak having the features of suddenness and uncertainty, highlight the need for improvements in global outbreak surveillance, strong public health systems are essential for maintaining and improving health and well-being [4], [5]. Timely collection and analysis of epidemiological information is helpful for making rapid decisions and responding to an infectious-disease emergency. It is thus necessary to have an unobstructed channel and convenient tool for the collection and analysis of epidemiologic information in the field.

With the development of communications, the Internet, intelligent mobile-phone services and WebGIS, new technologies have been widely used in the domain of the prevention and control of infectious diseases, use of unstructured event-based reports, internet media report and XML forms for infectious disease reporting and surveillance is a new application [6]–[8]. In recent years, surveillance systems of infectious diseases, such as GPHIN, Argus, HealthMap, and BioacaCaster, have been established for the collection, management, and analysis of epidemiologic data, which has promoted the prevention and control of infectious diseases [9]–[11]. Establishing decision support system for the response to infectious disease emergencies and using personal digital assistants (PDAs) and other mobile devices can be very effective improving data collection time and quality [12]. In Sri Lanka, a mobile phone-based infectious disease surveillance system was built to obtain animal health information from field veterinarians in a timely fashion to establish baseline patterns in animal health, and in United Kindom another mobile-phone based system was built to aid critical information transfer among policy makers and veterinarians and poultry staff for highly pathogenic avian influenza (HPAI) [13]–[14]. Resources for infection prevention and control on the World Wide Web were abundant and easily available [15]. Web GIS (Geographic Information System) have always shared many of the foundational data and enable remixing and repurposing those data, many scientists have utilized this technology for infectious disease surveillance and data collection [16]–[18]. Intelligent mobile-phone services and WebGIS have been accessible and inexpensive tool in our lives.

In recent years, there have been outbreaks of several emerging infectious diseases in China; i.e., the SARS epidemic in 2003 [19], the hand, foot and mouth disease since 2008 [20], influenza A (H1N1) in 2009 and a novel bunyavirus infection in 2010 [21]. There have also been frequent natural disasters; i.e., the Wenchuan earthquake in 2008 and the Yushu earthquake and Zhouqu mudslides in 2010. The epidemics of infectious diseases and natural disasters seriously threatened human health and safety, highlighting the importance of establishing tools for timely data collection and analysis of epidemiological information for rapid decision making and response to infectious-disease emergencies and natural disasters in the field. Yang et al. created an emergency reporting system for infectious-disease surveillance following the Wenchuan earthquake in 2008; the cases reported using mobile phones accounted for as much as 52.9% of all cases reported weekly from 19 May to 13 July in affected areas, demonstrating that the mobile phone is a useful communication tool for infectious-disease surveillance in areas hit by natural disasters [22]. In the influenza A (H1N1) epidemic in 2009, many systems about global surveillance, network analysis and data collection for influenza A (H1N1) cases were created [23]–[25]. However, besides the collection and communication of epidemiologic information in the field, it is important to analyze the data to support decision making, and to simulate the evolution of the infectious disease emergency in time to respond to emergencies. Considering the case of China, the current study aims to provide a flexible and efficient tool that can be used in the response to infectious-disease emergencies in the field, and to establish a Decision Support System for Response to Infectious Disease Emergencies (DSSRIDE) based on WebGIS and mobile services. There were two online interfaces for accessing the system (http://www.geoepi.com for personal computer (PC) users and http://m.geoepi.com for mobile-phone users. DSSRIDE is a semi-open system, and used in infectious disease surveillance and response to emergencies industry, users who want using this system could contact the administrator of the system and send E-mail to yapin0215@163.com to sign a “User service agreement” and obtain user name and password. The permission of the user would describe in the agreement.

## Materials and Methods

### Data Collection and Integration

We collected baseline information including the spatial distribution of notifiable infectious diseases and information relating to natural foci, hosts, vectors, medical services, the population census, and the characteristics of the geographical environment, transportation and climate for each county in mainland China. These data were obtained from the Chinese Center for Disease Control and Prevention, the National Bureau of Statistics of China, the National Meteorological Bureau of China, and projects of natural foci investigation [26]–[28]. A database was established by geo-coding the information on a digital map of county boundaries for the entire country.

### System Structure and Function Design of DSSRIDE

**Figure 1:**
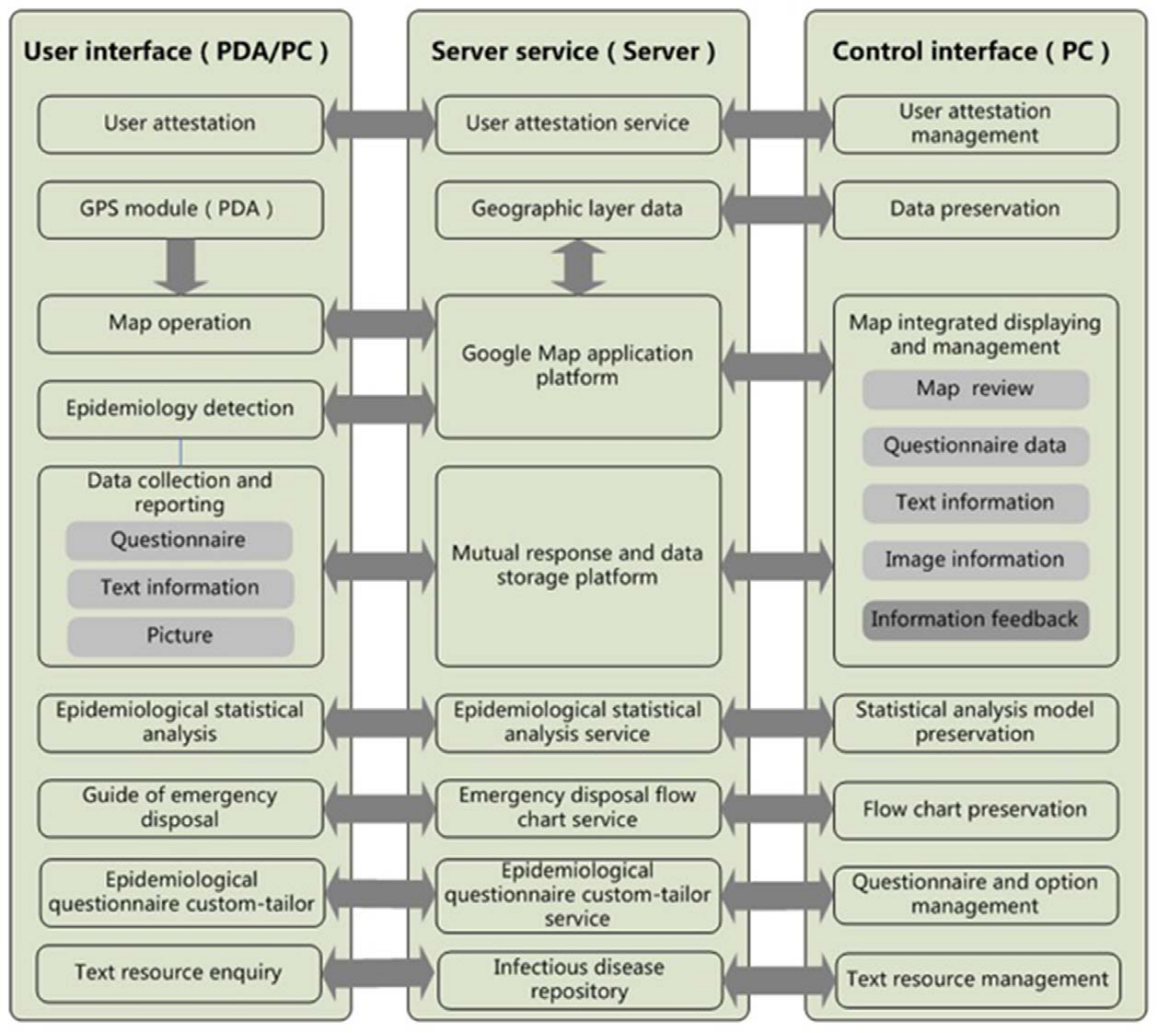
The system structure of the DSSRIDE. A B/S (Browser/Server) structure was used. General users access the interface by PC, PDA or mobile phone in the field, the server service provides data and map services to the users, and the control interface is accessed by the super administrator to add or delete data.

The system structure of the DSSRIDE comprised a user interface, server service and control interface with a B/S (Browser/Server) structure. The user interface included the functions and permissions and could be accessed by PC, PDA or mobile phone. Additionally, the location information in fields was obtained by keyword enquiry or Global Positioning System (GPS) tracking, and the function of the DSSRIDE was then performed according to the location information. The server service provides a map and data service. In this study, Google Maps was used to display geographic information and to make calculations related to maps. In addition, a control interface was designed for the manager of the system; administrative users were permitted to carry out actions such as creating or deleting usernames, and adding or revising data of the system (Figure 1).

### Design of the Function of the DSSRIDE

The system was mainly designed with four modules of function. Firstly, the system was designed to collect epidemiologic data in the field and to send the data to a database via a wireless Internet service. Secondly, an enquiry module was designed to display the baseline information of the spatial distribution of infectious diseases, natural foci, hosts, vectors, medical services, and population, and the characteristics of the geographic environment, transportation and climate. In addition, a customized epidemiologic questionnaire was designed for users to use in real time and online. Lastly, a query tool for handling different types of public health emergencies and obtaining associated professional knowledge was designed to assist medical workers and decision makers.

## Results

### Data Collection and Analysis

**Figure 2:**
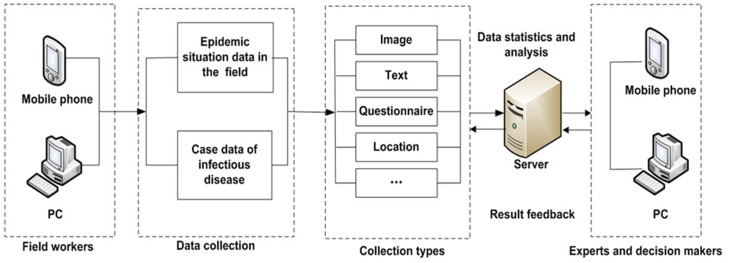
Flow chart of data collection, transmission, and information feedback between medical workers in the field and decision makers. Medical workers collect epidemiological information of patients, contact persons, the epidemic situation and locations by PC or PDA, and then send the data to the server. Experts analyze the data and provide feedback to workers in the field to guide their work in the field in real time.

Information including questionnaire results, text, pictures, voice recordings and video recorded in the field can be collected and sent to the database via wireless internet services from PCs, PDAs and mobile phones in real time by different users. Experts and officials can analyze the data to understand the dynamics of the infectious disease emergency, the temporal and spatial distributions of patients, the population at risk, the susceptible population, and probable influencing factors. Figure 2 shows the procedure of field investigation and data reporting. Information feedback and guides for medical workers in the field can also be sent easily.

More than 200 types of questionnaires including individual-case questionnaires and epidemic-case questionnaires are provided by the DSSRIDE. The system also provides epidemiological questionnaires customized according to users’ actual demands in the field. In addition, the geographic location is important for field investigations. In the DSSRIDE, location information is collected by a GPS unit or keyword enquiry, and the information of longitude and latitude is then integrated into the report data. The spatial distribution maps, figures of the epidemic curve, and patterns of gender, age and occupation can be created quickly, and epidemiologic analyses are conducted in real time. Additionally, users can export these additional data to an Excel spreadsheet for further epidemiology analysis in professional software, such as SPSS or STATA, which are not included in the DSSRIDE system.

### Epidemiological Detection

**Figure 3:**
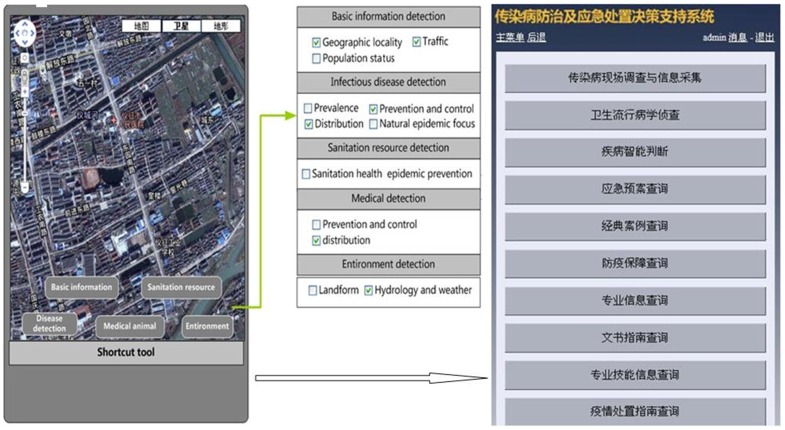
Epidemiological detection using PDA interfaces. Function design of epidemiological detection for the PDA client and application interface.

To understand the transmission risk of infectious diseases in decision making during emergencies, the baseline information of infectious diseases, hosts, vectors, medical services, the geographic environment and climate in the field is needed, and it is important to raise the efficiency of handling emergencies. This information can be determined from the location information in the DSSRIDE. Queries and spatial research can be carried out using Google Maps on a PC, PDA or mobile phone. Figure 3 shows the flow diagram of epidemiology analysis on a PDA and the application interface.

### Epidemiological Questionnaire Custom-tailor

**Figure 4:**
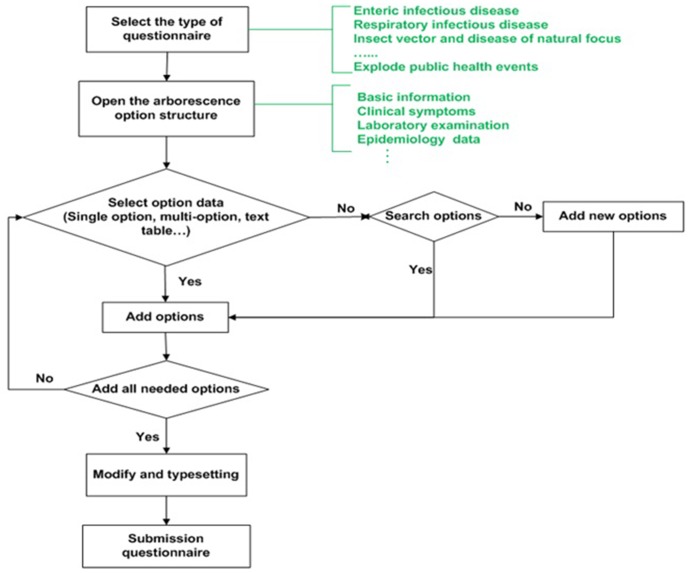
Flow chart of the questionnaire custom-tailor. The DSSRIDE provides more than 200 types of epidemiological questionnaires for users, and questionnaire custom-tailor is a new function for users in special emergency environments.

The DSSRIDE provides 10 categories of 206 epidemiological questionnaires for users. The system also provides the function that novel questionnaires can be customized in the field by users. Administrative users have the permission to manage questionnaires, including new questionnaires that are customized and optimized versions of existing questionnaires. Common users have the permission to put in, browse, modify, enquire about, and analyze questionnaire data and to export the information in a table format. Investigation and disposal workers can use the existing questionnaires or create novel questionnaires using the custom-tailor according to their needs in the field. Options for filling out questionnaires are readily provided by the system, and include uni-selections, multiple-selections, text, tables, and times. Users can choose the format of the questionnaire according to whether they are using a PC or PDA, and they are able to fill out, send, or review questionnaires online. The data of reported questionnaires are written to the database directly. Permitted users can analyze the data and transmit the results to the investigation worker in real time. Figure 4 shows the work flow of the questionnaire custom-tailor.

### Guides to Handling an Infectious Disease Emergency and Professional Knowledge Querying

The DSSRIDE integrates flow diagrams for the handling of an emergency, including handling cases of severe infectious diseases, emerging infectious diseases, food poisoning, nuclear, chemical and biologic hazards and other public health emergencies. Disposal workers and decision makers can be assisted by the flow diagrams in different emergencies, and can also be provided with information relating to the emergency, information for the prevention and control of infectious diseases, information relating to laboratory diagnosis, information relating to law, and information relating to medical services, disinfection, insecticides and sterilization, and case analyses.

### User Settings and Permission Management

The users of the DSSRIDE are classified into super administrators, administrators and general users. These users have the same permissions in terms of epidemiological detection, epidemiological statistical analysis, guidance for handling infectious disease and professional knowledge enquiries. However, there are different permissions for epidemiological investigations in the field and data collection. Besides having the permissions of the administrator, the super administrator can create an administrator and add, modify and delete data in the system. The administrator can create a general user and browse the data reported by the general users.

### Application of the DSSRIDE in the 2009 Pandemic Influenza A (H1N1) Epidemic in China

**Figure 5:**
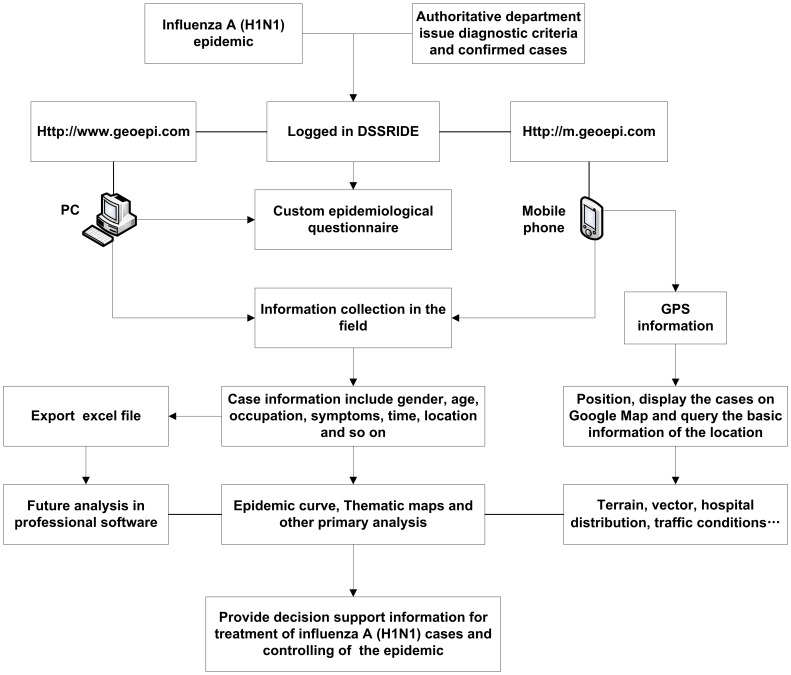
Application of the DSSRIDE in the prevention and control of the influenza A (H1N1) epidemic in 2009.

**Figure 6:**
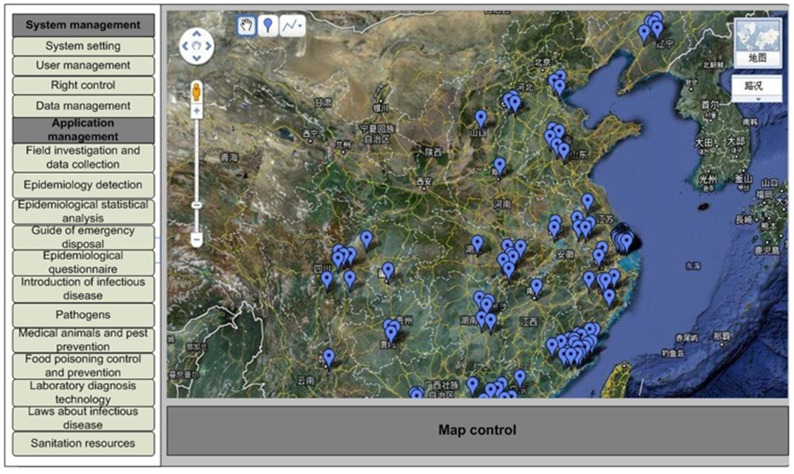
Data display and analysis in real time for the 2009 influenza A (H1N1) outbreak in mainland China. PC interface of the DSSRIDE and distribution of influenza A (H1N1) cases in the early stage of the epidemic in mainland China.

**Figure 7:**
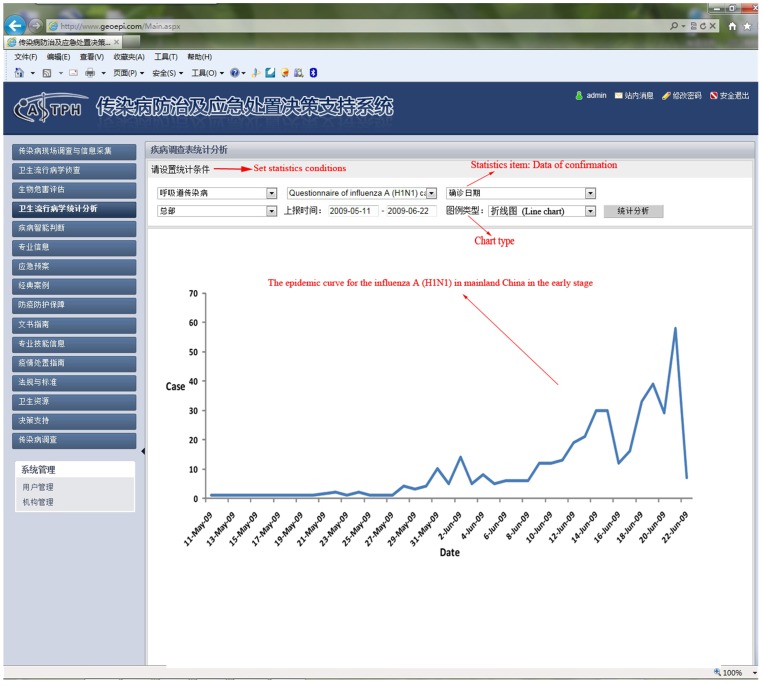
The epidemic curve for the influenza A (H1N1) in mainland China in the early stage from May 10 to June 22.

**Figure 8:**
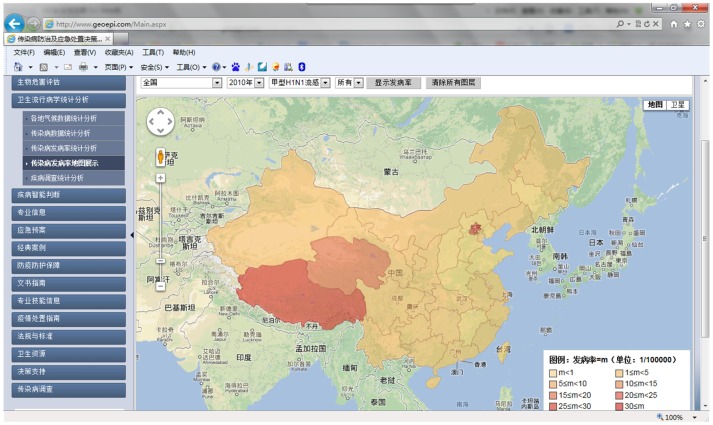
Hypsometric thematic map of the incidence of influenza A (H1N1) in different provinces in mainland China. We see the incidence of influenza A (H1N1) in different provinces in mainland China.

**Figure 9:**
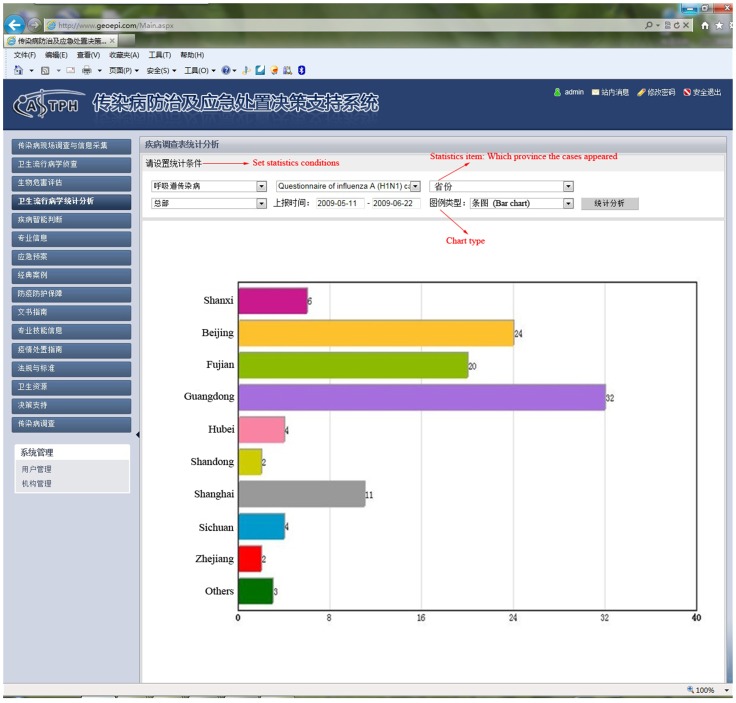
Bar chart of the distribution of influenza A (H1N1) cases in different provinces in early stage of the epidemic in the DSSRIDE. Using the DSSRIDE, users could analyze the collected data in real time and display them in various ways; e.g., as a bar chart, pie chart, line chart, or thematic map.

**Figure 10:**
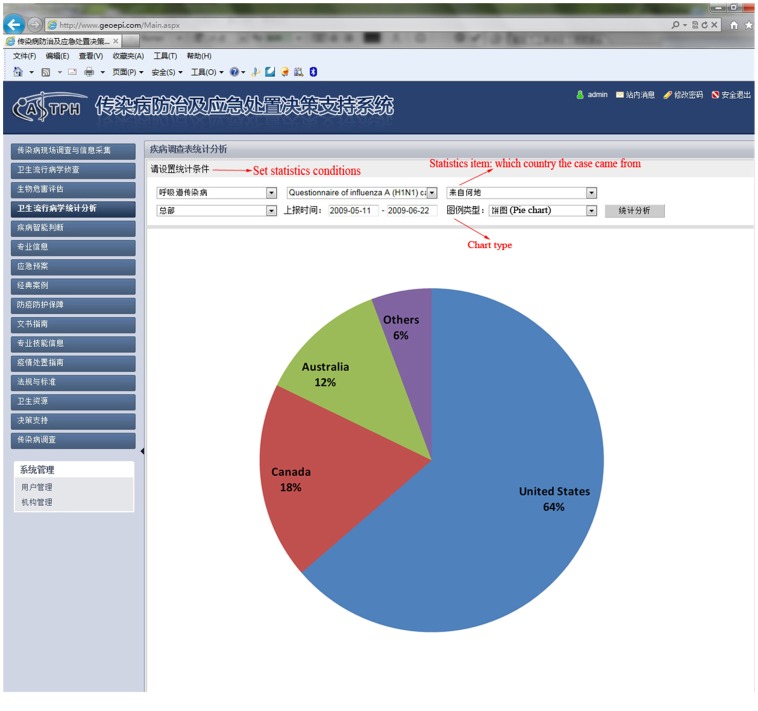
In the early stage of influenza A (H1N1) in mainland China, most of the cases were imported cases and came from United States, Canada and Australia. This pie showed the constituent ratio of these cases.

In 2009, pandemic influenza A (H1N1) emerged and spread globally. We collected information relating to cases in the early stage of the epidemic in mainland China from May 10 to June 22 using the DSSRIDE and analyzed the characteristics of these cases. Figure 5 illustrates the use of the DSSRIDE in this epidemic and presents the functions of the DSSRIDE. The influenza A (H1N1) cases were confirmed by authorities in accordance with diagnostic criteria published by the ministry of health of the People’s Republic of China. First, we customized a new questionnaire to collect related information on influenza A (H1N1) cases, including age, gender, occupation, symptoms, and location. Maps and epidemic curves for the temporal and spatial distributions of pandemic influenza A (H1N1) were then created, we can also displaying the incidence of influenza A (H1N1) by thematic map in DSSRIDE. (Figure 6, 7 and 8) In the early stage of the epidemic in mainland China, most of the cases appeared in Guangdong, Fujian, Shanghai and Beijing, the uses can statistics and display it by bar chart easily using DSSRIDE. (Figure 9) In the early stage, most of the influenza A (H1N1) cases were imported cases and probably 86% came from United States, Canada and Australia, the pie chart in DSSRIDE displaying it clearly. (Figure 10) The investigators in the field and the decision-makers in the office could understand the temperature, humidity, traffic conditions and distribution of hospitals for the locations of the cases, and the information was useful for patient treatment and the control of the epidemic. The information on the cases was exported and analysis was later carried out in Excel software; the results show that 77.8% of pandemic influenza A (H1N1) cases were imported cases, and 90% of the imported cases came from America, Canada and Australia from May 11 to June 22, 2009. The cases were distributed in 22 provinces, and 68.6% of the cases were in southern China. The mean incubation period was 4.2 (4.2±1.5) days and most patients had clinical symptoms including fever (81%), coughing (40%), and a sore throat (35%) (Tables 1 and 2) [29].

**Table 1:** Age–sex distribution for the influenza A (H1N1) cases in the early stage of the epidemic in mainland China.

Gender-Age	Total cases (n = 420)	Imported cases (n = 297)	Second-generation cases (n = 85)	*P*
Gender				
Male/Female(cases)	187/169 (45/40)	161/136 (54/46)	34/46 (43/57)	0.06
Age(year)	22±15(0.5–73)	23±15 (0.5–73)	19±14 (1.5–59)	
Age distribution				
0∼	16 (3.8)	13 (4.4)	3 (3.5)	
6∼	116 (27.6)	83 (27.9)	33 (38.8)	
15∼	113 (26.9)	96 (32.3)	17 (20.0)	
25∼	40 (9.5)	29 (9.8)	10 (11.8)	
35∼	26 (6.2)	21 (7.1)	5 (5.9)	
45∼	31 (7.4)	27 (9.1)	4 (4.7)	
55∼	10 (2.4)	8 (2.7)	2 (2.4)	
65∼	4 (1.0)	4 (1.3)	–	
Unknown	64 (15.2)	16 (5.4)	9 (10.6)	

Note: Comparison between imported and second-generation cases, *P = *0.06.

**Table 2:** Clinical characteristics for the influenza A (H1N1) cases in the early stage of the epidemic in mainland China.

Symptom	Cases			χ^2*a*^	P
	Total cases (N = 420)	Imported cases(N = 297)	Second-generation cases (N = 85)		
Fever	341 (81.2)	264 (88.9)	65 (76.5)	8.53	0.00
Cough	168 (40.0)	108 (36.4)	49 (57.6)	12.37	0.00
Sore throat	148 (35.2)	85 (28.6)	52 (61.2)	30.45	0.00
Runny nose	41 (9.7)	35 (11.8)	6 (7.1)	1.54	0.21
Headache	23 (5.5)	19 (6.4)	4 (4.7)	0.33	0.56
Fatigue	23 (5.2)	19 (6.4)	4 (4.7)	0.33	0.56
Muscle aches and pains	18 (4.3)	15 (5.0)	3 (3.5)	0.08	0.77
Expectoration	9 (2.1)	6 (2.0)	3 (3.5)	0.16	0.69
Chills	5 (1.0)	5 (1.7)	0	-	0.59
Vomit	3 (0.7)	2 (0.7)	1 (1.2)	-	0.53
Chest tightness	2 (0.5)	2 (0.7)	0	-	1.00
dizziness	3 (0.5)	2 (0.7)	4 (4.7)	4.59	0.03
Diarrhea	2 (0.5)	1 (0.3)	1 (1.2)	0.89	0.34
Syncope	1 (0.2)	1 (0.3)	0	-	1.00

Note: The data in the brackets are incidences (%); ***^a^*** Comparison between imported and second-generation cases; - Fisher’s exact test.

## Discussion

This study established a decision support system for response to infectious disease emergencies using WebGIS and mobile services (DSSRIDE). The DSSRIDE provides functions including data collection, communication and analyses in real time, epidemiological detection, the provision of customized epidemiological questionnaires and guides for handling infectious disease emergencies, and professional knowledge querying in the field. These functions of the DSSRIDE could be helpful for epidemiological investigation in the field and the disposal of materials in an infectious disease emergency.

The DSSRIDE is an integrated decision support system to aid the response to infectious disease emergencies and it is accessed easily by PCs, PDAs and mobile phones. It is thus suitable for use in the field, which is its main difference when compared with other information systems of infectious disease surveillance. In addition, most surveillance systems of infectious disease focus on data collection, communication, and display; e.g., the surveillance system for infectious diseases of pets in Chile and the surveillance systems for the distribution of mosquito species and global HPAI [14], [30]–[32]. The Chinese Center for Disease Prevention and Control established a mobile phone-based surveillance system as an emergency reporting system for infectious diseases [22]. The DSSRIDE is not only a data collection and transmission system; users can also communicate with each other, query professional knowledge in the field and consult with experts in offices in real time. In addition, the questionnaire custom-tailor provides more than 200 commonly used questionnaires and allows the use of customized, novel questionnaires by users in the field. Administrative users have permission to customize novel questionnaires in the field. Investigation workers in the field fill out and send questionnaires online, and the information is transmitted to the server in real time. This is a very convenient and direct method of making information available. The baseline information, including that relating to infectious diseases, hosts, vectors, medical services, the geographic environment and climate throughout the country, could help public health workers enhance the efficiency of handling emergencies and increase the chances of successfully controlling an emerging infectious disease. Users can search for the position that an emergency takes place using the Google Maps platform on a PC or PDA, and obtain professional knowledge. Users can characterize and display such information using bar graphs, pie charts, and line charts. These functions increase the availability of specialized knowledge and provide information support for emergency disposal workers and decision makers. The establishment and application of the DSSRIDE could provide new methods of data collection and analysis of infectious diseases in real time in the field, and the system will improve the ability to respond to infectious disease emergencies. Using the relatively simple analysis functions of the system, users can export questionnaire data as an Excel file and analyze them in professional analysis software such as SPSS and STATA. All the investigation data are saved by the system, and if third parties want to share the data, we can act as an intermediary to coordinate the sharing of data.

The successful application of the DSSRIDE requires investments including those in software and hardware. There are three types of hardware: the data and map server, PCs, and mobile phones (including PDAs). The servers are used to run the DSSRIDE and provide the service to the users. Users use PCs, mobile phones and PDAs to access the system to collect, transmit, browse and analyze data in the field and in offices. In the market, many brands and models of mobile phones support access of the DSSRIDE; e.g., Nokia, Motorola, HTC, and Blackberry. Considering the low cost of application, normal-configuration hardware is suitable for using the DSSRIDE, and low-income districts can be included in the system. The interface and database of the DSSRIDE were designed in Chinese, and apply to Chinese language districts and populations. The system is designed flexibly, and it can be easily adapted to not only the response to animal infectious disease emergences but also to the response to bioterrorism, earthquakes, flood and other natural disasters.

### Conclusion

The DSSRIDE is a decision support system used in infectious disease emergencies. The system includes functions for field investigations, data collection, transmission and analyses in real time, epidemiological detection, customizing questionnaires, and querying professional knowledge. The DSSRIDE provides a geographic information platform based on the Google Maps application programming interface to display information of infectious disease emergencies, and transfers information between workers in the field and decision makers through wireless transmission based on PCs, mobile phones and PDAs. Users can gain the baseline information at sites of emergencies, such as the baseline information of infectious diseases, hosts, vectors, medical services, geographic environment and climate. The DSSRIDE is simple, flexible and cheap, and is suitable to use in developing countries and low-income districts. The DSSRIDE can be used by different users such as international organizations, nations and provinces, hospitals, and survey groups. The DSSRIDE can easily be extended to adapt other domains such as animal infectious diseases, plant diseases and insect pests in agriculture, bioterrorism, earthquakes, floods and other natural disasters. It could be helpful for handling infectious disease emergencies or other public health events.

### Ethics and Patient Confidentiality Issues for the DSSRIDE

Ethics is a key problem in the system application, and we attach great importance to this issue. The users of the system may be health agencies, hospitals, temporary teams of investigators or other agencies or organizations. Before such groups begin using the system, we will sign an agreement with the appropriate ethics committee to avoid ethical problems.

The patient’s private information must be kept confidential. Anyone wanting to use the data must be reviewed by the appropriate ethical committee and sign a confidentiality agreement. All individuals need to comply with the relevant laws and regulations in using the data.
